# *N*′-(2-Chloro­benzyl­idene)benzo­hydrazide

**DOI:** 10.1107/S1600536809040331

**Published:** 2009-10-10

**Authors:** Chuan-Gang Fan, Ming-Zhi Song

**Affiliations:** a College of Chemistry and Chemical Technology, Binzhou University, Binzhou 256600, Shandong, People’s Republic of China

## Abstract

The asymmetric unit of the title compound, C_14_H_11_ClN_2_O, contains two independent mol­ecules. In one mol­ecule, the two aromatic rings form a dihedral angle of 45.94 (16)°, while in the second mol­ecule this angle is 58.48 (16)°. In the crystal, inter­molecular N—H⋯O hydrogen bonds link the mol­ecules into two crystallographically independent sets of chains propagating along [001].

## Related literature

For the biological properties of Schiff base ligands, see: Bedia *et al.* (2006 ▶). For related crystal structures, see: Fun *et al.* (2008 ▶); Alhadi *et al.* (2008 ▶); Nie (2008 ▶).
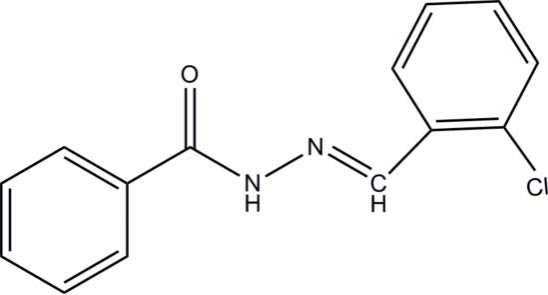


## Experimental

### 

#### Crystal data

C_14_H_11_ClN_2_O
*M* *_r_* = 258.70
Tetragonal, 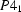

*a* = 13.5588 (17) Å
*c* = 14.3993 (18) Å
*V* = 2647.2 (6) Å^3^
*Z* = 8
Mo *K*α radiation
μ = 0.28 mm^−1^
*T* = 298 K
0.45 × 0.42 × 0.38 mm

#### Data collection

Bruker SMART APEX CCD area-detector diffractometer
Absorption correction: multi-scan (*SADABS*; Sheldrick, 1996 ▶) *T*_min_ = 0.885, *T*_max_ = 0.902
10838 measured reflections
4614 independent reflections
2766 reflections with *I* > 2σ(*I*)
*R*_int_ = 0.043

#### Refinement

*R*[*F* ^2^ > 2σ(*F* ^2^)] = 0.041
*wR*(*F* ^2^) = 0.093
*S* = 1.03
4614 reflections
325 parameters
1 restraint
H-atom parameters constrained
Δρ_max_ = 0.21 e Å^−3^
Δρ_min_ = −0.23 e Å^−3^
Absolute structure: Flack (1983 ▶), 2105 Friedel pairs
Flack parameter: −0.03 (7)

### 

Data collection: *SMART* (Siemens, 1996 ▶); cell refinement: *SAINT* (Siemens, 1996 ▶); data reduction: *SAINT*; program(s) used to solve structure: *SHELXS97* (Sheldrick, 2008 ▶); program(s) used to refine structure: *SHELXL97* (Sheldrick, 2008 ▶); molecular graphics: *SHELXTL* (Sheldrick, 2008 ▶); software used to prepare material for publication: *SHELXTL*.

## Supplementary Material

Crystal structure: contains datablocks I, global. DOI: 10.1107/S1600536809040331/cv2620sup1.cif

Structure factors: contains datablocks I. DOI: 10.1107/S1600536809040331/cv2620Isup2.hkl

Additional supplementary materials:  crystallographic information; 3D view; checkCIF report

## Figures and Tables

**Table 1:** Hydrogen-bond geometry (Å, °)

*D*—H⋯*A*	*D*—H	H⋯*A*	*D*⋯*A*	*D*—H⋯*A*
N1—H1⋯O1^i^	0.86	2.01	2.854 (4)	168
N3—H3⋯O2^ii^	0.86	2.09	2.928 (4)	166

Symmetry codes: (i) 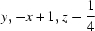; (ii) 
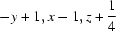
.

**Fig. 1.**
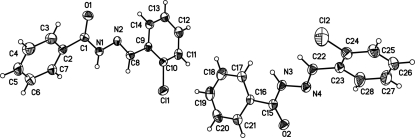
The content of asymmetric unit of the title compound showing the atomic numbering scheme and 30% probability displacement ellipsoids.

## supplementary crystallographic information

### Comment

Schiff base ligands have received considerable attention during the last decades, mainly because of diversity of their structures and biological properties (Bedia *et al.*, 2006). We report here the crystal structure of the title new Schiff base compound (I).

In (I) (Fig. 1), the bond lengths and angles are normal and comparable with those observed in similar compounds (Nie *et al.*, 2008; Fun *et al.*, 2008; Alhadi *et al.*, 2008). The asymmetric unit of (I) contains two independent molecules- *A* and *B*, respectively. In molecule *A*, two aromatic rings form a dihedral angle of 45.94 (16)°, while in molecule *B* this angle is 58.48 (16) °.

In the crystal structure, intermolecular N—H···O hydrogen bonds (Table 1) link molecules into two crystallographically independent sets of chains propagated in direction [001].

### Experimental

Benzohydrazide (5.0 mmol),  ethanol (20 ml) and 2-chlorobenzaldehyde (5.0 mmol) were mixed in 50 ml flash. After refluxing for 3 h, the resulting mixture was cooled to room temperature, and recrystalized from ethanol, and afforded the title compound as a crystalline solid. Elemental analysis: calculated for C_14_H_11Cl_N_2_O: C 65.00, H 4.29, N 10.83%; found: C 65.13, H 4.36, N 10.74%.

### Refinement

All H atoms were placed in geometrically idealized positions (N—H 0.86 Å, C—H 0.93 Å) and treated as riding on their parent atoms, with *U*_iso_(H) = 1.2*U*_eq_(C) (C,*N*).

### Crystal data

**Table:**

C_14_H_11_ClN_2_O	*D*_x_ = 1.298 Mg m^−^^3^
*M**_r_* = 258.70	Mo *K*α radiation, λ = 0.71073 Å
Tetragonal, *P*4_1_	Cell parameters from 2946 reflections
*a* = 13.5588 (17) Å	θ = 2.6–25.3°
*c* = 14.3993 (18) Å	µ = 0.28 mm^−^^1^
*V* = 2647.2 (6) Å^3^	*T* = 298 K
*Z* = 8	Needle, colourless
*F*(000) = 1072	0.45 × 0.42 × 0.38 mm

### Data collection

**Table:**

Bruker SMART APEX CCD area-detector diffractometer	4614 independent reflections
Radiation source: fine-focus sealed tube	2766 reflections with *I* > 2σ(*I*)
graphite	*R*_int_ = 0.043
φ and ω scans	θ_max_ = 25.0°, θ_min_ = 1.5°
Absorption correction: multi-scan (*SADABS*; Sheldrick, 1996)	*h* = −13→16
*T*_min_ = 0.885, *T*_max_ = 0.902	*k* = −6→16
10838 measured reflections	*l* = −16→17

### Refinement

**Table:**

Refinement on *F*^2^	Secondary atom site location: difference Fourier map
Least-squares matrix: full	Hydrogen site location: inferred from neighbouring sites
*R*[*F*^2^ > 2σ(*F*^2^)] = 0.041	H-atom parameters constrained
*wR*(*F*^2^) = 0.093	*w* = 1/[σ^2^(*F*_o_^2^) + (0.0242*P*)^2^ + 0.8271*P*] where *P* = (*F*_o_^2^ + 2*F*_c_^2^)/3
*S* = 1.03	(Δ/σ)_max_ < 0.001
4614 reflections	Δρ_max_ = 0.21 e Å^−^^3^
325 parameters	Δρ_min_ = −0.23 e Å^−^^3^
1 restraint	Absolute structure: Flack (1983), 2105 Friedel pairs
Primary atom site location: structure-invariant direct methods	Flack parameter: −0.03 (7)

### Special details

**Table:**

Geometry. All e.s.d.'s (except the e.s.d. in the dihedral angle between two l.s. planes) are estimated using the full covariance matrix. The cell e.s.d.'s are taken into account individually in the estimation of e.s.d.'s in distances, angles and torsion angles; correlations between e.s.d.'s in cell parameters are only used when they are defined by crystal symmetry. An approximate (isotropic) treatment of cell e.s.d.'s is used for estimating e.s.d.'s involving l.s. planes.
Refinement. Refinement of *F*^2^ against ALL reflections. The weighted *R*-factor *wR* and goodness of fit *S* are based on *F*^2^, conventional *R*-factors *R* are based on *F*, with *F* set to zero for negative *F*^2^. The threshold expression of *F*^2^ > σ(*F*^2^) is used only for calculating *R*-factors(gt) *etc*. and is not relevant to the choice of reflections for refinement. *R*-factors based on *F*^2^ are statistically about twice as large as those based on *F*, and *R*- factors based on ALL data will be even larger.

### Fractional atomic coordinates and isotropic or equivalent isotropic displacement parameters (Å^2^)

**Table:**

	*x*	*y*	*z*	*U*_iso_*/*U*_eq_	
Cl1	0.75607 (8)	0.65234 (7)	0.24623 (8)	0.0633 (3)	
Cl2	0.88744 (9)	−0.28276 (9)	0.02864 (13)	0.1075 (6)	
N1	0.4131 (2)	0.5479 (2)	0.3296 (2)	0.0408 (8)	
H1	0.4139	0.5829	0.2798	0.049*	
N2	0.4972 (2)	0.53797 (19)	0.3835 (2)	0.0405 (8)	
N3	0.9519 (2)	0.0803 (2)	0.0223 (2)	0.0495 (9)	
H3	0.9112	0.0700	0.0670	0.059*	
N4	1.0075 (2)	0.0039 (2)	−0.0142 (2)	0.0494 (8)	
O1	0.32675 (18)	0.44465 (18)	0.42320 (19)	0.0536 (7)	
O2	1.00890 (19)	0.18599 (17)	−0.0864 (2)	0.0543 (7)	
C1	0.3299 (3)	0.5015 (3)	0.3567 (3)	0.0402 (9)	
C2	0.2392 (2)	0.5252 (3)	0.3029 (3)	0.0418 (9)	
C3	0.1631 (3)	0.4574 (3)	0.3042 (3)	0.0642 (13)	
H3A	0.1708	0.3979	0.3354	0.077*	
C4	0.0752 (3)	0.4782 (4)	0.2591 (3)	0.0827 (15)	
H4	0.0250	0.4314	0.2577	0.099*	
C5	0.0623 (3)	0.5671 (4)	0.2167 (4)	0.0843 (16)	
H5	0.0022	0.5819	0.1888	0.101*	
C6	0.1368 (3)	0.6345 (4)	0.2148 (3)	0.0742 (14)	
H6	0.1278	0.6944	0.1845	0.089*	
C7	0.2262 (3)	0.6142 (3)	0.2578 (3)	0.0542 (10)	
H7	0.2770	0.6602	0.2564	0.065*	
C8	0.5738 (3)	0.5796 (3)	0.3501 (3)	0.0425 (9)	
H8	0.5704	0.6102	0.2924	0.051*	
C9	0.6667 (2)	0.5797 (2)	0.4014 (3)	0.0399 (9)	
C10	0.7535 (3)	0.6149 (2)	0.3604 (3)	0.0411 (9)	
C11	0.8398 (3)	0.6218 (3)	0.4122 (3)	0.0536 (11)	
H11	0.8971	0.6458	0.3848	0.064*	
C12	0.8404 (3)	0.5931 (3)	0.5037 (4)	0.0599 (11)	
H12	0.8980	0.5986	0.5383	0.072*	
C13	0.7562 (3)	0.5561 (3)	0.5445 (3)	0.0551 (11)	
H13	0.7571	0.5351	0.6060	0.066*	
C14	0.6715 (3)	0.5508 (3)	0.4937 (3)	0.0485 (10)	
H14	0.6147	0.5268	0.5220	0.058*	
C15	0.9631 (2)	0.1712 (3)	−0.0140 (3)	0.0429 (9)	
C16	0.9164 (2)	0.2533 (2)	0.0384 (3)	0.0382 (9)	
C17	0.8908 (3)	0.2462 (3)	0.1306 (3)	0.0509 (11)	
H17	0.9009	0.1873	0.1623	0.061*	
C18	0.8502 (3)	0.3259 (3)	0.1762 (3)	0.0638 (13)	
H18	0.8329	0.3207	0.2385	0.077*	
C19	0.8352 (3)	0.4131 (3)	0.1296 (4)	0.0666 (14)	
H19	0.8080	0.4668	0.1605	0.080*	
C20	0.8601 (3)	0.4213 (3)	0.0382 (4)	0.0624 (13)	
H20	0.8499	0.4805	0.0070	0.075*	
C21	0.9006 (2)	0.3416 (3)	−0.0083 (3)	0.0481 (10)	
H21	0.9172	0.3471	−0.0708	0.058*	
C22	0.9846 (3)	−0.0815 (3)	0.0139 (3)	0.0517 (11)	
H22	0.9300	−0.0898	0.0521	0.062*	
C23	1.0434 (3)	−0.1671 (3)	−0.0132 (3)	0.0534 (11)	
C24	1.0065 (3)	−0.2622 (3)	−0.0086 (3)	0.0586 (11)	
C25	1.0630 (4)	−0.3424 (3)	−0.0335 (3)	0.0702 (14)	
H25	1.0364	−0.4055	−0.0300	0.084*	
C26	1.1573 (4)	−0.3296 (3)	−0.0630 (3)	0.0798 (15)	
H26	1.1956	−0.3839	−0.0791	0.096*	
C27	1.1960 (4)	−0.2360 (4)	−0.0689 (4)	0.0951 (19)	
H27	1.2604	−0.2270	−0.0896	0.114*	
C28	1.1396 (3)	−0.1556 (3)	−0.0444 (4)	0.0887 (18)	
H28	1.1664	−0.0926	−0.0487	0.106*	

### Atomic displacement parameters (Å^2^)

**Table:**

	*U*^11^	*U*^22^	*U*^33^	*U*^12^	*U*^13^	*U*^23^
Cl1	0.0684 (7)	0.0568 (6)	0.0646 (7)	−0.0023 (5)	0.0190 (6)	0.0062 (6)
Cl2	0.0785 (9)	0.0592 (8)	0.1849 (18)	−0.0126 (7)	0.0274 (10)	0.0013 (9)
N1	0.0326 (18)	0.049 (2)	0.041 (2)	−0.0026 (15)	−0.0042 (15)	0.0040 (15)
N2	0.0316 (17)	0.0436 (18)	0.046 (2)	−0.0003 (15)	−0.0021 (16)	−0.0020 (15)
N3	0.053 (2)	0.0354 (18)	0.060 (2)	0.0021 (15)	0.0208 (17)	−0.0004 (16)
N4	0.0535 (19)	0.0383 (18)	0.057 (2)	0.0034 (16)	0.0091 (17)	−0.0023 (17)
O1	0.0470 (16)	0.0590 (18)	0.055 (2)	−0.0098 (13)	−0.0089 (13)	0.0145 (15)
O2	0.0614 (18)	0.0442 (16)	0.0573 (19)	−0.0025 (14)	0.0186 (16)	0.0003 (14)
C1	0.042 (2)	0.038 (2)	0.040 (2)	−0.0029 (18)	−0.0036 (19)	0.0017 (19)
C2	0.033 (2)	0.052 (2)	0.040 (2)	−0.0049 (19)	−0.0028 (18)	−0.0031 (19)
C3	0.049 (3)	0.079 (3)	0.065 (3)	−0.018 (2)	−0.010 (2)	0.011 (3)
C4	0.049 (3)	0.125 (4)	0.074 (4)	−0.029 (3)	−0.015 (3)	0.012 (3)
C5	0.039 (3)	0.140 (5)	0.073 (4)	0.008 (3)	−0.010 (2)	0.011 (4)
C6	0.062 (3)	0.089 (4)	0.071 (4)	0.017 (3)	−0.009 (3)	0.013 (3)
C7	0.042 (2)	0.066 (3)	0.054 (3)	0.003 (2)	−0.002 (2)	0.003 (2)
C8	0.043 (2)	0.043 (2)	0.041 (2)	0.0016 (19)	0.0004 (19)	0.0003 (19)
C9	0.033 (2)	0.034 (2)	0.052 (3)	−0.0017 (17)	0.0039 (19)	−0.0032 (19)
C10	0.047 (2)	0.030 (2)	0.047 (3)	−0.0011 (18)	0.010 (2)	−0.0036 (18)
C11	0.040 (2)	0.041 (2)	0.080 (4)	−0.0033 (18)	0.006 (2)	−0.006 (2)
C12	0.045 (3)	0.059 (3)	0.076 (4)	−0.002 (2)	−0.009 (3)	−0.006 (3)
C13	0.044 (3)	0.067 (3)	0.054 (3)	0.000 (2)	−0.004 (2)	0.004 (2)
C14	0.038 (2)	0.053 (2)	0.054 (3)	−0.0016 (18)	−0.001 (2)	0.005 (2)
C15	0.035 (2)	0.038 (2)	0.056 (3)	−0.0038 (17)	0.002 (2)	−0.002 (2)
C16	0.032 (2)	0.033 (2)	0.050 (3)	−0.0007 (16)	0.0021 (17)	−0.0049 (19)
C17	0.047 (2)	0.048 (2)	0.058 (3)	0.003 (2)	−0.001 (2)	−0.003 (2)
C18	0.066 (3)	0.063 (3)	0.062 (3)	0.000 (3)	0.008 (2)	−0.022 (3)
C19	0.054 (3)	0.056 (3)	0.090 (4)	0.005 (2)	−0.002 (3)	−0.027 (3)
C20	0.053 (3)	0.036 (2)	0.098 (4)	0.003 (2)	−0.016 (3)	−0.001 (3)
C21	0.043 (2)	0.038 (2)	0.063 (3)	−0.0031 (18)	−0.001 (2)	−0.001 (2)
C22	0.054 (2)	0.039 (2)	0.062 (3)	0.001 (2)	0.017 (2)	−0.001 (2)
C23	0.057 (3)	0.044 (3)	0.059 (3)	0.010 (2)	0.012 (2)	0.006 (2)
C24	0.068 (3)	0.041 (2)	0.067 (3)	0.003 (2)	0.005 (3)	−0.001 (2)
C25	0.091 (4)	0.048 (3)	0.072 (4)	0.015 (3)	0.003 (3)	−0.002 (2)
C26	0.100 (4)	0.059 (3)	0.081 (4)	0.034 (3)	0.017 (3)	0.006 (3)
C27	0.086 (4)	0.070 (4)	0.130 (5)	0.017 (3)	0.047 (4)	0.015 (3)
C28	0.076 (3)	0.055 (3)	0.136 (5)	0.006 (3)	0.043 (3)	0.012 (3)

### Geometric parameters (Å, °)

**Table:**

Cl1—C10	1.721 (4)	C11—H11	0.9300
Cl2—C24	1.724 (4)	C12—C13	1.379 (5)
N1—C1	1.350 (4)	C12—H12	0.9300
N1—N2	1.386 (4)	C13—C14	1.364 (5)
N1—H1	0.8600	C13—H13	0.9300
N2—C8	1.277 (4)	C14—H14	0.9300
N3—C15	1.347 (4)	C15—C16	1.487 (5)
N3—N4	1.385 (4)	C16—C17	1.375 (5)
N3—H3	0.8600	C16—C21	1.389 (5)
N4—C22	1.264 (4)	C17—C18	1.379 (5)
O1—C1	1.230 (4)	C17—H17	0.9300
O2—C15	1.230 (4)	C18—C19	1.375 (6)
C1—C2	1.488 (5)	C18—H18	0.9300
C2—C3	1.382 (5)	C19—C20	1.364 (6)
C2—C7	1.381 (5)	C19—H19	0.9300
C3—C4	1.387 (5)	C20—C21	1.385 (5)
C3—H3A	0.9300	C20—H20	0.9300
C4—C5	1.362 (6)	C21—H21	0.9300
C4—H4	0.9300	C22—C23	1.461 (5)
C5—C6	1.362 (6)	C22—H22	0.9300
C5—H5	0.9300	C23—C24	1.385 (5)
C6—C7	1.389 (5)	C23—C28	1.388 (5)
C6—H6	0.9300	C24—C25	1.376 (5)
C7—H7	0.9300	C25—C26	1.359 (6)
C8—C9	1.459 (5)	C25—H25	0.9300
C8—H8	0.9300	C26—C27	1.376 (6)
C9—C14	1.388 (5)	C26—H26	0.9300
C9—C10	1.400 (5)	C27—C28	1.378 (6)
C10—C11	1.390 (5)	C27—H27	0.9300
C11—C12	1.374 (6)	C28—H28	0.9300
			
C1—N1—N2	118.7 (3)	C12—C13—H13	120.4
C1—N1—H1	120.6	C13—C14—C9	122.6 (4)
N2—N1—H1	120.6	C13—C14—H14	118.7
C8—N2—N1	114.5 (3)	C9—C14—H14	118.7
C15—N3—N4	118.4 (3)	O2—C15—N3	122.4 (3)
C15—N3—H3	120.8	O2—C15—C16	121.5 (3)
N4—N3—H3	120.8	N3—C15—C16	116.1 (4)
C22—N4—N3	115.5 (3)	C17—C16—C21	119.3 (4)
O1—C1—N1	123.1 (3)	C17—C16—C15	123.0 (3)
O1—C1—C2	120.8 (3)	C21—C16—C15	117.7 (4)
N1—C1—C2	116.1 (3)	C16—C17—C18	120.3 (4)
C3—C2—C7	119.4 (3)	C16—C17—H17	119.8
C3—C2—C1	117.8 (3)	C18—C17—H17	119.8
C7—C2—C1	122.6 (3)	C19—C18—C17	120.0 (5)
C2—C3—C4	120.0 (4)	C19—C18—H18	120.0
C2—C3—H3A	120.0	C17—C18—H18	120.0
C4—C3—H3A	120.0	C20—C19—C18	120.3 (4)
C5—C4—C3	120.0 (4)	C20—C19—H19	119.9
C5—C4—H4	120.0	C18—C19—H19	119.9
C3—C4—H4	120.0	C19—C20—C21	120.1 (4)
C6—C5—C4	120.5 (4)	C19—C20—H20	120.0
C6—C5—H5	119.8	C21—C20—H20	120.0
C4—C5—H5	119.8	C20—C21—C16	120.0 (4)
C5—C6—C7	120.3 (5)	C20—C21—H21	120.0
C5—C6—H6	119.8	C16—C21—H21	120.0
C7—C6—H6	119.8	N4—C22—C23	120.5 (4)
C2—C7—C6	119.6 (4)	N4—C22—H22	119.7
C2—C7—H7	120.2	C23—C22—H22	119.7
C6—C7—H7	120.2	C24—C23—C28	117.4 (4)
N2—C8—C9	120.8 (3)	C24—C23—C22	122.0 (4)
N2—C8—H8	119.6	C28—C23—C22	120.6 (4)
C9—C8—H8	119.6	C25—C24—C23	121.5 (4)
C14—C9—C10	117.4 (3)	C25—C24—Cl2	118.3 (3)
C14—C9—C8	121.7 (3)	C23—C24—Cl2	120.2 (3)
C10—C9—C8	120.8 (4)	C26—C25—C24	120.3 (4)
C11—C10—C9	120.3 (4)	C26—C25—H25	119.9
C11—C10—Cl1	118.4 (3)	C24—C25—H25	119.9
C9—C10—Cl1	121.3 (3)	C25—C26—C27	119.7 (4)
C12—C11—C10	120.0 (4)	C25—C26—H26	120.1
C12—C11—H11	120.0	C27—C26—H26	120.1
C10—C11—H11	120.0	C26—C27—C28	120.2 (5)
C11—C12—C13	120.5 (4)	C26—C27—H27	119.9
C11—C12—H12	119.8	C28—C27—H27	119.9
C13—C12—H12	119.8	C27—C28—C23	121.0 (4)
C14—C13—C12	119.2 (4)	C27—C28—H28	119.5
C14—C13—H13	120.4	C23—C28—H28	119.5

### Hydrogen-bond geometry (Å, °)

**Table:**

*D*—H···*A*	*D*—H	H···*A*	*D*···*A*	*D*—H···*A*
N1—H1···O1^i^	0.86	2.01	2.854 (4)	168
N3—H3···O2^ii^	0.86	2.09	2.928 (4)	166

Symmetry codes: (i) *y*, −*x*+1, *z*−1/4; (ii) −*y*+1, *x*−1, *z*+1/4.
